# Effect of salt reduction on iodine status assessed by 24 hour urinary iodine excretion in children and their families in northern China: a substudy of a cluster randomised controlled trial

**DOI:** 10.1136/bmjopen-2016-011168

**Published:** 2016-09-08

**Authors:** Feng J He, Yuan Ma, Xiangxian Feng, Wanqi Zhang, Laixiang Lin, Xiaohui Guo, Jing Zhang, Wenyi Niu, Yangfeng Wu, Graham A MacGregor

**Affiliations:** 1 Wolfson Institute of Preventive Medicine, Barts and The London School of Medicine & Dentistry, Queen Mary University of London, London, UK; 2 The George Institute for Global Health at Peking University Health Science Center, Beijing, China; 3 Department of Epidemiology and Biostatistics, Peking University School of Public Health, Beijing, China; 4 Changzhi Medical College, Shanxi, China; 5 School of Public Health, Tianjin Medical University, Tianjin, China; 6 Key Laboratory of Hormone and Development (Ministry of Health), Tianjin, China; 7 Metabolic Diseases Hospital & Tianjin Institute of Endocrinology, Tianjin Medical University, Tianjin, China; 8 Department of Social Medicine and Health Education, Peking University School of Public Health, Beijing, China; 9 Peking University Clinical Research Institute, Beijing, China

**Keywords:** Salt reduction, Iodine status, 24h urine collections, Cluster randomised trial

## Abstract

**Objective:**

To study the effect of salt reduction on iodine status and to determine whether iodine consumption was still adequate after salt reduction in a population where universal salt iodisation is mandatory.

**Design:**

A substudy of a cluster randomised controlled trial, with schools randomly assigned to either the intervention or the control group.

**Setting:**

28 primary schools in Changzhi, northern China.

**Participants:**

279 children in grade 5 of primary school (mean age: 10.1); 553 adults (age: 43.8).

**Intervention:**

Children were educated about the harmful effects of salt and how to reduce salt intake using the schools' usual health education lessons. Children then delivered the message to their families. The duration was 1 school term (≈3.5 months).

**Main outcome measure:**

Difference between the intervention and control groups in the change of iodine intake as measured by repeat 24 hour urinary iodine from baseline to the end of the trial.

**Results:**

At baseline, the mean salt intake was 7.0±2.5 g/day in children and 11.7±4.4 g/day in adults and the median iodine intake was 165.1 μg/day (IQR: 122.6–216.7) and 280.7 μg/day (IQR: 205.1–380.9) in children and adults, respectively. At the end of the study, salt and iodine decreased in the intervention compared with control group. The mean effect on salt for intervention versus control was −1.9 g/day (95% CI −2.6 to −1.3) in children and −2.9 g/day (95% CI −3.7 to −2.2) in adults. The mean effect on iodine was −19.3% (95% CI −29.4% to −7.7%) in children and −11.4% (95% CI −20.3% to −1.5%) in adults.

**Conclusions:**

With ≈25% reduction in salt intake, there was a significant reduction in iodine consumption in northern China where salt is iodised. Despite this, iodine intake was still adequate, and well above the estimated average requirement. Our findings indicate that reducing salt to the WHO's target—30% reduction by 2025—will not compromise iodine status.

**Trial registration number:**

ClinicalTrials.gov NCT01821144.

**Strengths and limitations of this study**

- Twenty-four hour urinary iodine excretion is the most reliable biochemical marker for assessing iodine status.
- Our study is the first to have assessed iodine status by repeat 24 hour urine collections in a large number of primary schoolchildren and their adult family members in northern China where universal salt iodisation is mandatory.
- Our study, for the first time, has assessed the effect of a modest reduction in salt intake on iodine status using a well-controlled randomised trial.
- The results demonstrate that ≈25% reduction in salt intake, which is close to the WHO's target, does not compromise iodine status.
- Despite all 24 hour urine collections followed stringent protocol with careful supervision, there might still be under-collections in some participants. However, the consistent findings from various sensitivity analyses indicate that this is unlikely to alter the primary outcome.

## Introduction

Iodine deficiency disorder is a global public health problem with ∼1.88 billion people, including 241 million school-age children, having insufficient intake of iodine worldwide.^1^ China was one of the countries that had a serious epidemic of iodine deficiency disorders.^2^ In 1993, the WHO and Unicef recommended universal salt iodisation to prevent and control iodine deficiency.^1^ China launched a universal salt iodisation programme in 1995.^3^ Since then significant progress has been made in reducing iodine deficiency disorders.^3^ ^4^ In recent years, there has been debate about the optimal levels of iodine fortification in salt, particularly as salt intake is very high in China and iodine excess could also lead to thyroid diseases.^3^ ^5^ ^6^

A reduction in salt intake is one of the most cost-effective public health policies to prevent hypertension and cardiovascular disease.^7–9^ The WHO recommends a 30% reduction in salt intake by 2025 for all countries around the world with an eventual target of 5 g/day.^10^ As salt has been used as a vehicle for iodine fortification in many countries, it is important to monitor iodine status to ensure that iodine consumption is still adequate when salt intake is reduced.

More than 90% of iodine consumed is excreted in the urine within 24–48 hours.^11^ ^12^ Therefore, 24 hour urinary iodine excretion is a good marker of recent dietary iodine intake and is the ideal biochemical indicator for assessing iodine status.^1^ We measured 24 hour urinary iodine excretion in individuals who took part in School-EduSalt (*School*-based *Edu*cation Programme to Reduce *Salt*),^13^ ^14^ a cluster randomised controlled trial in Changzhi, northern China where universal salt iodisation is mandatory. The primary aim of the School-EduSalt trial was to determine whether an education programme targeted at primary schoolchildren could lower salt intake in children and their families. The study collected two consecutive 24 hour urines at baseline and at the end of the trial using a standardised protocol with careful supervision. The results showed that the education led to a significant reduction in salt intake by ∼25% in children and adults compared with the controls. In this article, we report a prespecified substudy,^15^ the aim of which was to assess iodine status by repeat 24 hour urinary iodine excretion and to study the effect of salt reduction on iodine status, and in particular to determine whether iodine consumption was still adequate after the participants had been on a reduced salt intake for a few months.

## Methods

A detailed description of the methods of the School-EduSalt study has been published elsewhere^13^ ^14^ and the abridged methods are reported here. The study was a cluster randomised controlled trial in 28 primary schools in urban Changzhi, Northern China. From each school, we selected one class in grade 5 (age ≈10 years). From each class, we randomly selected 10 children who met the inclusion criteria.^14^ From each child's family, we also enrolled two adults. Schools were randomly assigned to either the intervention or the control group with stratification by the location of schools and the size of the class.

Children in the intervention group were educated about the harmful effects of salt on health and how to reduce salt intake using the schools' usual health education lessons, that is, one 40 min lesson every 2 weeks.^13^ ^14^ The salt reduction education was delivered to the whole class in spite of only 10 children being selected for assessment. Children were asked to deliver the salt reduction message to the families, particularly children needed to persuade the persons who did the cooking to reduce the amount of salt used during food preparation at home. The duration of the intervention was one school term (≈3.5 months). Children in the control group carried on with their usual health education lessons as in the curriculum.

The primary outcome of this substudy was the difference between the intervention and the control group in the change of iodine intake as measured by 24 hour urinary iodine excretion from baseline to the end of the trial.

Urinary iodine was measured by the Key Laboratory of Hormone and Development (Ministry of Health, China) that participated in the US Centers for Disease Control and Prevention EQUIP (Ensuring the Quality of Urinary Iodine Procedures) programme.^16^ Ammonium persulfate digestion with spectrophotometric detection of the Sandell-Kolthoff reaction was used for urinary iodine measurement with quality control,^17^ using the samples collected during the study with the storage condition of −80°C. For each batch of samples, we ran four levels of certified reference material—lyophilised human urine (lot numbers GBW09108l, GBW09110n, GBW09111a and GBW09112a; National Reference Laboratory for iodine deficiency disorder, Beijing) with mean certified iodine concentrations of 67.9 μg/L (95% CI 58.9 to 76.9), 195 μg/L (95% CI 185 to 205), 558 μg/L (95% CI 541 to 575) and 885 μg/L (95% CI 857 to 913), respectively. The biochemists who performed the urinary iodine measurements were not aware which group the participant was allocated.

### Statistical analyses

As urinary iodine was not normally distributed, we used median and IQR to summarise the iodine status. Three urine samples with iodine >5000 μg/24 hour were outliers and excluded from the analysis. All three were from the intervention group. We used the cut-off points (EAR, estimated average requirement and UL, tolerable upper limit) as recommended by the Chinese Nutrition Society^18^ to define iodine intake as insufficient if urinary iodine was less than EAR, that is, <65 μg/24 hour in children aged ≈10 or <85 μg/24 hour in adults; adequate if iodine was between EAR and UL, that is, 65–300 μg/24 hour in children or 85–600 μg/24 hour in adults; and excessive if urinary iodine was more than UL, that is, >300 μg/24 hour in children or >600 μg/24 hour in adults. For the purpose of comparison with other surveys, we also reported 24 hour urinary iodine concentration and iodine status based on urinary iodine concentration according the WHO's criteria (ie, iodine deficient <100 μg/L; adequate 100–199 μg/L, above requirement 200–299 μg/L; excessive ≥300 μg/L).

Our main analysis was based on intention to treat using linear mixed models as reported previously.^14^ ^19^ Logarithmic transformed iodine was used, and as such, the mean effect on iodine was presented as percentage change. The statistical model was in the form: Outcome=Group+Time+Interaction (time×group)+Stratification variables at randomisation (school location and class size)+Confounding variables (age, sex, body mass index, indoor and outdoor temperature). To examine the robustness of the conclusions of the primary analysis, we carried out various sensitivity analyses as specified previously.^14^ The number of 24 hour urine samples included and excluded in each analysis was shown in online supplementary figure 1.

10.1136/bmjopen-2016-011168.supp2 Supplementary figure Trial profile. ITT: Intention-to-treat.

We used SAS (V.9.4) for the analyses. Results are reported as mean, SD and 95% CI or median and IQR where appropriate. All analyses were two sided and p values of <0.05 were considered statistically significant.

## Results

The School-EduSalt trial enrolled 279 children and 553 adults, all of whom were included in the current report. The baseline characteristics of the participants were well balanced between the intervention and the control group (see online supplementary table 1). The mean age was 10.1±0.5 years for children and 43.8±12.2 years for adults.

10.1136/bmjopen-2016-011168.supp1 Supplementary table

The result on salt has been published previously.^14^ We report it again in this article explicitly for the purpose of allowing the readers to compare the salt and iodine levels. At baseline, the mean salt intake as calculated from 24 hour urinary sodium excretion was 7.0±2.5 g/day in children and 11.7±4.4 g/day in adults. The median iodine consumption as measured by 24 hour urinary iodine was 165.1 µg/day (IQR: 122.6–216.7, 95% CI 156.9 to 172.9) and 280.7 µg/day (IQR: 205.1–380.9, 95% CI 270.3 to 293.8) in children and adults, respectively.

Table 1 shows the salt and iodine intake by group, as well as their changes during the study. From baseline to the end of the trial, salt and iodine intake decreased in the intervention group and increased in the control group. The mean effect size on salt for intervention versus control was −1.9 g/day (95% CI −2.6 to −1.3, p<0.0001) in children and −2.9 g/day (95% CI −3.7 to −2.2, p<0.0001) in adults. The mean effect size on iodine was −19.3% (95% CI −29.4% to −7.7%, p=0.002) in children and −11.4% (95% CI −20.3% to −1.5%, p=0.03) in adults.

**Table 1:** Salt and iodine intake as calculated from 24 hour urinary sodium and iodine excretion based on intention-to-treat analysis

	Control			Intervention				
Outcome	Baseline*	End of trial*	Change from baseline*	Baseline*	End of trial*	Change from baseline*	Mean effect† (intervention vs control)	p Value
*Children*								
Salt mean‡ (95% CI) (g/day)	6.8 (6.2 to 7.4)	8.0 (7.4 to 8.6)	1.2 (0.7 to 1.7)	7.3 (6.7 to 7.9)	6.6 (6.0 to 7.2)	−0.7 (−1.2 to −0.2)	−1.9 (−2.6 to −1.3)	<0.0001
Iodine								
Geometric mean (95% CI) (µg/day)	162.8 (146.7 to 180.5)	187.5 (168.9 to 208.0)	115.2% (104.7% to 126.7%)	173.7 (156.7 to 192.4)	163.2 (147.2 to 180.9)	94.0% (85.6% to 103.2%)	−19.3% (−29.4% to −7.7%)	0.002
Median (IQR) (µg/day)	161.7 (117.7 to 209.5)	176.0 (136.5 to 237.2)	27.4 (−18.3 to 76.7)	167.0 (128.9 to 217.7)	154.8 (118.6 to 234.1)	−13.1 (−54.5 to 37.8)		
*Adults*								
Salt mean (95% CI) (g/day)	11.3 (10.5 to 12.1)	12.1 (11.3 to 12.9)	0.8 (0.2 to 1.3)	12.6 (11.8 to 13.3)	10.4 (9.7 to 11.2)	−2.1 (−2.7 to −1.6)	−2.9 (−3.7 to −2.2)	<0.0001
Iodine								
Geometric mean (95% CI) (µg/day)	271.2 (245.1 to 300.1)	284.6 (256.9 to 315.2)	104.9% (97.2% to 113.3%)	291.2 (263.3 to 322.1)	271.9 (245.7 to 301.0)	93.4% (86.6% to 100.7%)	−11.4% (−20.3% to −1.5%)	0.030
Median (IQR) (µg/day)	262.1 (197.8 to 357.5)	281.3 (207.9 to 387.6)	10.7 (−72.8 to 105.3)	297.4 (213.2 to 390.8)	258.5 (199.8 to 350.0)	−36.5 (−128.4 to 88.9)		

*Mean and geometric mean were adjusted for stratification variables at randomisation (school location and class size).

†Adjusted for age, sex, body mass index, stratification variables at randomisation (school location and class size), and indoor and outdoor temperature.

‡The results for salt were taken from previous report.^14^

Table 2 shows iodine status according to the Chinese Nutrition Society's guidelines.^18^ In the intervention group, there was an increase in the proportion of individuals with iodine intake below EAR from baseline to the end of the trial. Despite this, there were only <5% children and <3% adults who had iodine intake below EAR after salt intake was reduced.

**Table 2:** Iodine status assessed by 24 hour urinary iodine excretion

	Control		Intervention	
Category	Baseline, N (%)	End of trial, N (%)	Baseline, N (%)	End of trial, N (%)
Children				
<65 (µg/day) (EAR)	5 (3.62)	1 (0.74)	1 (0.71)	6 (4.32)
65–300 (µg/day)	123 (89.13)	114 (84.44)	128 (90.78)	119 (85.61)
>300 (µg/day) (UL)	10 (7.25)	20 (14.81)	12 (8.51)	14 (10.07)
Adults				
<85 (µg/day) (EAR)	3 (1.09)	4 (1.53)	2 (0.72)	7 (2.58)
85–600 (µg/day)	260 (94.55)	243 (93.10)	263 (94.95)	243 (89.67)
>600 (µg/day) (UL)	12 (4.36)	14 (5.36)	12 (4.33)	21 (7.75)

EAR, estimated average requirement; UL, tolerable upper limit.

The results from sensitivity analyses are shown in online supplementary table 2. The first analysis excluded possibly incomplete 24 hour urine collections. As expected, the absolute levels of salt and iodine intake were higher compared with those when all 24 hour urine collections were included. However, the primary outcome, that is, the difference between the two groups in the change of salt and iodine intake, was very similar to that from the main analysis. The results for completers (ie, the participants who had 24 hour urine collections at baseline and end of the trial) and per-protocol analyses (including completers with complete 24 hour urine collections) were very close to those from the corresponding analyses with all participants included.

Online supplementary table 3 shows the iodine status based on 24 hour urinary iodine concentration using the WHO's criteria, as well as the median 24 hour urinary iodine concentration and the median 24 hour urinary iodine excretion for each category. In children and adults, the median 24 hour urinary iodine excretions in the group classified as iodine deficient according to the WHO's criteria (ie, <100 μg/L) were well above EAR across the study.

## Discussion

**Figure 1:**
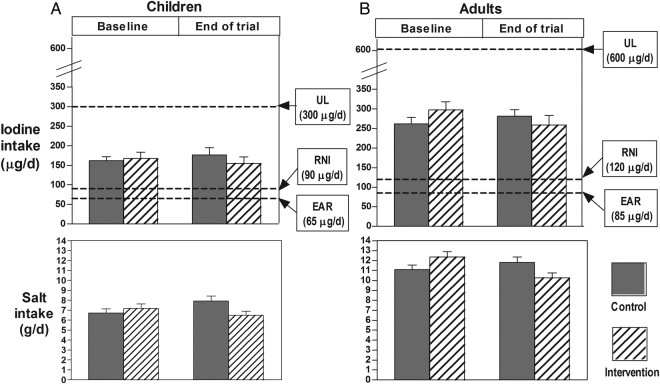
Mean salt, median iodine intake and their 95% CIs in children (A) and adults (B). EAR, estimated average requirement; RNI, recommended nutrient intake; UL, tolerable upper limit.

Our study produced two important findings. First, the study for the first time has measured iodine intake using repeat 24 hour urine collections in a large number of primary schoolchildren and their families in northern China. A conservative estimate showed that the median baseline iodine intake was 165 µg/day in children and 281 µg/day in adults. These intakes are adequate. According to the Chinese Nutrition Society's guideline, EAR (ie, daily intake meeting the requirement of one-half of the population) is 65 µg/day in children aged 7–10 years and 85 µg/day in adults, and recommended nutrient intake (RNI, ie, intake meeting the requirement of 97–98% of the population) is 90 µg/day in children aged 7–10 and 120 µg/day in adults.^18^ The median iodine intakes in our study were 254% and 331% of EAR and 183% and 234% of RNI for children and adults, respectively. Additionally, the median iodine intakes were far below the UL of 300 µg/day in children and 600 µg/day in adults (figure 1).

Second, our study is the first to have studied the effect of salt reduction, as currently recommended, on iodine status in a population where salt is universally iodised. The mean effect was a reduction in salt intake of 1.9 g/day in children and 2.9 g/day in adults which led to a decrease in iodine intake of 19.3% and 11.4% in children and adults, respectively. These mean effects represent the differences between the intervention and control group in the changes in salt and iodine from baseline to the end of the trial. As shown in table 1, during the study, salt and iodine intake decreased in the intervention group and increased in the control group. If applying the mean reduction in iodine level (19.3% in children and 11.4% in adults) to all participants irrespective of their group allocation, the average iodine intake would be 133 μg/day in children and 249 μg/day in adults after salt reduction. These iodine levels are still adequate, and 205% and 293% of EAR and 148% and 208% of RNI for children and adults, respectively.

In our study, all 24 hour urine collections were carefully supervised with the start and finish time recorded by trained research staff. It is certain that there was no over-collection. However, it is difficult to know whether there was any under-collection. Although the participants who admitted to having missed urine voids were asked to re-do 24 hour urine collections, it is still possible that some participants did not report missing urine collection. Excluding potential incomplete 24 hour urine collections, as expected, led to a slightly higher salt and iodine intake for baseline and end trial, and for the intervention and the control group. It is therefore likely that our main results have under-estimated the average salt and iodine intake of the study population. However, this is unlikely to alter the primary outcome, that is, the difference between the intervention and control group. Indeed, various sensitivity analyses have shown consistent findings (see online supplementary table 2).

In Changzhi where our study was carried out, the iodine content in salt varied from 18 to 33 mg/kg in 2013 (data were provided by the local salt manufacturer). Based on the iodine content in salt and the 24 hour urinary sodium and iodine excretion, we estimated that ≈80% of iodine in the diet was from iodised salt. The changes in 24 hour urinary iodine observed in our study is consistent with that predicted from the changes in salt intake (see online supplementary table 4). Therefore, any potential influence from other dietary sources would be small.

Despite 24 hour urinary iodine is the most reliable biochemical marker for assessing iodine status, almost all previous surveys on iodine have used spot urine due to the apparent logistic challenges and costs in collecting 24 hour urine. The WHO also endorsed the use of spot urine and provided cut-offs of median spot urinary iodine concentration to categorise population's iodine status.^1^ However, this has been inappropriately used by previous surveys to define the number of individuals who were iodine deficient.^20^ Our study demonstrates that, in the group of individuals classified as iodine deficient according to the WHO's criteria based on urinary iodine concentration, the median 24 hour urinary iodine levels were well above EAR. These findings clearly illustrate the inappropriateness of spot urine in monitoring iodine status, and as a result, previous surveys would have over-estimated the prevalence of iodine deficiency. It is worth noting that our study did not collect spot urine; however, 24 hour urinary iodine concentration is a better index than any of the spot urine iodine concentration (eg, casual, first morning void). Additionally, our study shows that it is entirely feasible to collect 24 hour urine not only in adults but also in primary schoolchildren. The WHO has recommended 24 hour urine collections for determining and monitoring population salt intake.^21^ It will be more efficient and highly cost-effective if the iodine intake is monitored in the same population surveys using the same methods.

In China, since the introduction of universal salt iodisation in 1995, regular surveys using casual spot urine have been carried out to monitor the population's iodine status and adjust the iodine content in salt accordingly.^3^ The surveys were largely conducted in primary schoolchildren aged 8–10 because these children are readily accessible in schools and they have been assumed to have iodine intakes characteristic of general populations. At country level, the median spot urinary iodine in schoolchildren aged 8–10 increased from 165 μg/L in 1995 to over 300 μg/L by 1999 and declined to 241 and 246 μg/L in 2002 and 2005, respectively.^3^ This was in parallel with the changes of iodine content in salt which increased from 16.2 mg/kg in 1995 to 42.3 mg/kg in 1999, then declined to 30.8 mg/kg in 2005 and has remained at this level.^3^ These changes reflect the alterations of the standard for ‘qualified’ iodised salt set by the Chinese Ministry of Health.^3^ Initially the regulation for iodine content was ≥20 mg/kg in 1995. As there was no upper limit, most salt producers tended to iodise salt with iodine over 40 mg/kg. In 1997, an upper limit of 60 mg/kg was set. National iodine survey at the time indicated an excessive population iodine intake and such data led to a reduction in the upper limit from 60 to 50 mg/kg in 2002. The standard of 35±15 (or 20–50) mg/kg had remained until 2012 when provinces were allowed to choose from the three standards, that is, 20 (14–26), 25 (18–33) and 30 (21–39) mg/kg, depending on local diet and spot urinary iodine concentration.^22^

In our study site—Changzhi, the changes in urinary iodine followed a similar pattern to that occurred nationally although some of the surveys showed a higher iodine level. The most recent survey in Changzhi was carried out in 2010 and showed that the median spot urinary iodine was 241, 284 and 310 μg/L in schoolchildren aged 8, 9 and 10, respectively.^23^ In our study which was performed in 2013, the median baseline 24 hour urinary iodine concentration was 215.8 μg/L for schoolchildren aged ≈10 years. The lower iodine level observed in our study could be largely due to the decrease in iodine content in salt following the change in the standard for iodised salt (ie, from 20–50 to 18–33 mg/kg) in 2012.

Despite our study was carried out in Changzhi and included individuals who mainly ate home-made meals, the results could be broadly applicable to most parts of China for the following reasons: (1) universal salt iodisation is mandatory in China, and the food manufacturers and restaurants also use iodised salt; (2) the iodine content in salt (18–33 mg/kg) in Changzhi is similar to the national level (14–39 mg/kg);^22^ and (3) salt is the major source of iodine in the diet across China. Although there is a variation in iodine level from natural sources such as water and foods, iodised salt contributes to 60–80% of total iodine intake in most parts of China.^24^ ^25^ In Changzhi where our study was carried out, iodised salt accounts for ≈80% of iodine intake (ie, at the higher end of the range in China). The iodine intake in our study population was still adequate after an approximate 25% reduction in salt intake for 3.5 months; it is therefore most likely that the same reduction in salt if achieved across China would not compromise iodine status. The findings of our study, however, may not be generalisable to populations in other countries due to a number of features in the setting, such as universal salt iodisation and high contribution of discretionary salt to total salt intake in the Chinese diet.

## Conclusions

Our study demonstrates that in northern China where universal salt iodisation is mandatory, a reduction in salt intake by ≈25% which is close to the WHO's target of 30% reduction by 2025 does not compromise iodine status as measured by repeat 24 hour urinary iodine excretion in children and adults. These findings provide strong support for the WHO's recommendations to reduce population salt intake to prevent hypertension and cardiovascular disease, and to improve iodine intake by fortifying salt with iodine to prevent iodine deficiency.

Currently many countries have started salt reduction initiatives and also implemented salt iodisation programmes.^26^ However, there is a lack of coordination between the two. To maximise the benefits, there is an urgent need for close coordination and collaboration, particularly in disseminating consistent messages and monitoring population salt and iodine intake using the same methods which will provide valuable data required for appropriate adjustment of the iodine level in salt after population salt intake is reduced. This will be the most cost-effective way in implementing the two important public health policies.
