# Supplementary figures and images for: Effect of salt reduction on iodine status assessed by 24 hour urinary iodine excretion in children and their families in northern China: a substudy of a cluster randomised controlled trial
Source: BMJ Open. 2016 Sep 8;6(9):e011168. doi: 10.1136/bmjopen-2016-011168 (PMC5051323; doi:10.1136/bmjopen-2016-011168)

Supplement Figure 1. Trial profile. ITT: Intention-to-treat.

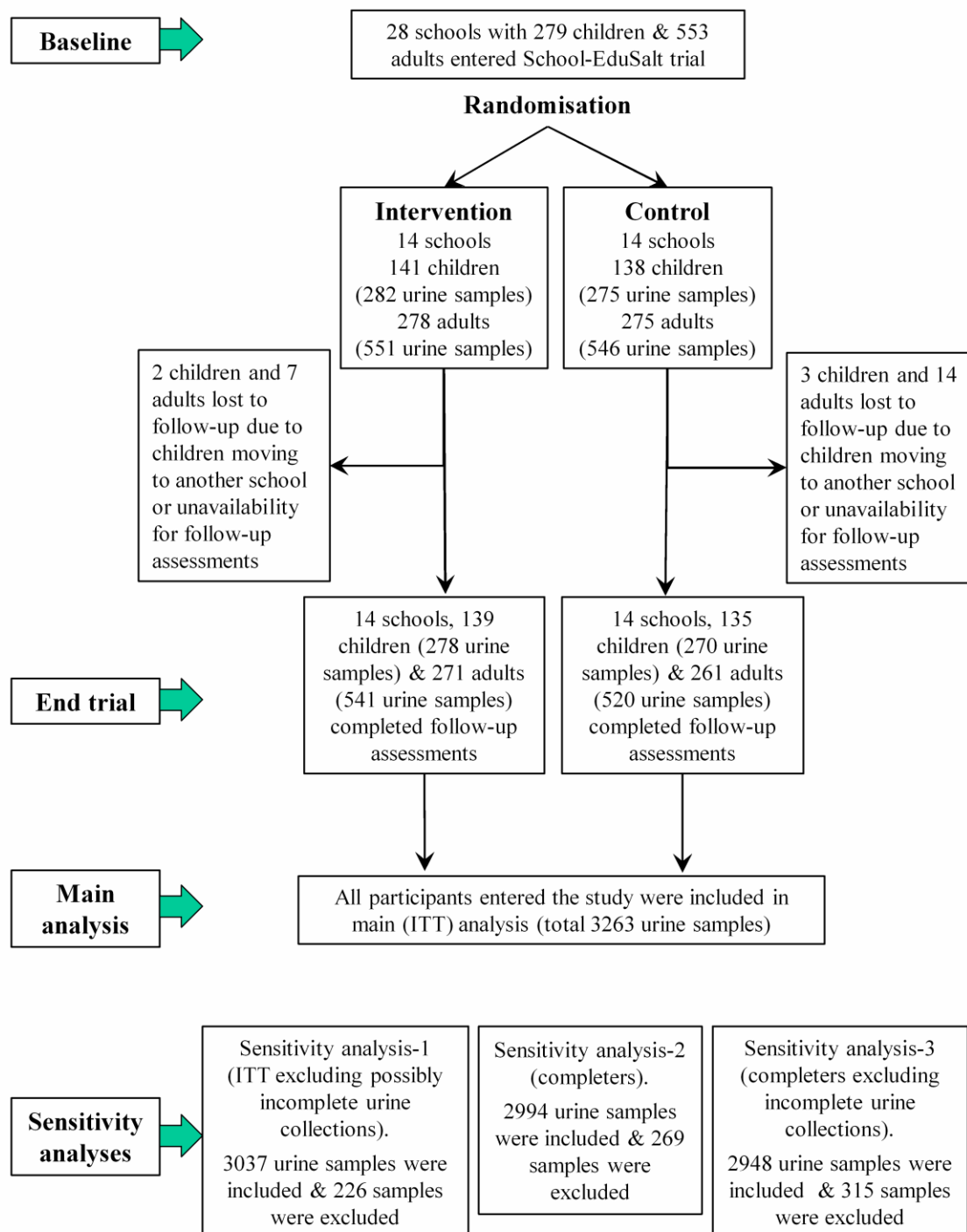

Supplement: Supplementary figure — Trial profile. ITT: Intention-to-treat. [file bmjopen-2016-011168supp_figure.pdf]
