# Supplementary material for: Effect of salt reduction on iodine status assessed by 24 hour urinary iodine excretion in children and their families in northern China: a substudy of a cluster randomised controlled trial
Source: BMJ Open. 2016 Sep 8;6(9):e011168. doi: 10.1136/bmjopen-2016-011168 (PMC5051323; doi:10.1136/bmjopen-2016-011168)
Supplement: Supplementary table [file bmjopen-2016-011168supp_tables.pdf]

ONLINE SUPPLEMENT

Effect of salt reduction on iodine status assessed by 24h urinary iodine excretion in children and their families in northern China: a sub-study of a cluster randomised controlled trial

Feng J He,[1] Yuan Ma,[1,2,3] Xiangxian Feng,[4] Wanqi Zhang,[5,6] Laixiang Lin,[6,7] Xiaohui Guo,[5] Jing Zhang,[2] Wenyi Niu,[8] Yangfeng Wu,[2,3,9] Graham A MacGregor[1]

[1] Wolfson Institute of Preventive Medicine, Barts and The London School of Medicine & Dentistry, Queen Mary University of London, UK.
[2] The George Institute for Global Health at Peking University Health Science Center, Beijing, China.
[3] Department of Epidemiology and Biostatistics, Peking University School of Public Health, Beijing, China.
[4] Changzhi Medical College, Shanxi, China.
[5] School of Public Health, Tianjin Medical University, Tianjin, China.
[6] Key Laboratory of Hormone and Development (Ministry of Health), China.
[7] Metabolic Diseases Hospital & Tianjin Institute of Endocrinology, Tianjin Medical University, Tianjin, China.
[8] Department of Social Medicine and Health Education, Peking University School of Public Health, Beijing, China.
[9] Peking University Clinical Research Institute, Beijing, China.

Correspondence to:
Dr. Feng J He
Wolfson Institute of Preventive Medicine, Barts and The London School of Medicine & Dentistry, Queen Mary University of London, Charterhouse Square, London EC1M 6BQ.
Tel: +44 (0)20 7882 6266
Fax: +44 (0)20 7882 6270
E-mail: f.he@qmul.ac.uk

Professor Wanqi Zhang
School of Public Health, Tianjin Medical University, Tianjin, China. 300070
Tel: +86 (0)22 8333 6595
Fax: +86 (0)22 8333 6608
E-mail: wqzhang@tmu.edu.cn

Supplement Table 1. Baseline characteristics of the participants*

Parameters	Control	Intervention	All
**Children**			
Number of schools	14	14	28
Number of children	138	141	279
Boys, N (%)	67 (48.6)	67 (47.5)	134 (48.0)
Age (year)	10.2 (0.5)	10.0 (0.5)	10.1 (0.5)
Weight (kg)	33.3 (7.2)	33.4 (7.8)	33.3 (7.5)
Height (cm)	140.7 (6.6)	139.2 (6.2)	140.0 (6.5)
Body mass index (kg/m^2)	16.7 (2.7)	17.1 (3.2)	16.9 (3.0)
**Adults**			
Number of adults	275	278	553
Men, N (%)	133 (48.4)	135 (48.6)	268 (48.5)
Parents, N (%)	208 (75.6)	203 (73.0)	411 (74.3)
Grandparents, N (%)	67 (24.4)	75 (27.0)	142 (25.7)
Age (year)	43.6 (11.8)	43.9 (12.5)	43.8 (12.2)
Weight (kg)	66.2 (12.9)	66.1 (11.6)	66.2 (12.3)
Height (cm)	162.8 (8.7)	162.4 (8.0)	162.6 (8.4)
Body mass index (kg/m^2)	24.9 (3.6)	25.0 (3.4)	24.9 (3.5)

*Data are means (SD) unless otherwise specified.

Supplement Table 2. Sensitivity analysis for salt and iodine intake as calculated from 24h urinary sodium and iodine excretion

Outcome	Number of participant Control /Intervention	Control		Intervention		Adjusted difference‖ (intervention vs control)	P value
		Baseline‡	Change from baseline‡	Baseline‡	Change from baseline‡		
**Population excluding possibly incomplete 24h urine**							
**Children**							
Salt (mean, 95%CI) (g/d)	138/140	7.0 (6.4—7.6)	1.2 (0.7—1.6)	7.5 (6.9—8.1)	-0.7 (-1.1 — -0.2)	-1.9 (-2.6— -1.2)	<0.0001
Iodine (geometric mean, 95%CI) (μg/d)	138/140	170.8 (155.0—188.2)	112.9% (102.7%—124.2%)	180.0 (163.6—198.1)	95.4% (86.9%—104.8%)	-16.5% (-26.9%— -4.6%)	0.008
Iodine (median, IQR) (μg/d)	138/140	166.5 (119.0—220.4)	27.3 (-20.6—79.0)	175.4 (131.3—228.7)	-9.3 (-52.4—39.0)		
**Adults**							
Salt (mean, 95%CI) (g/d)	273/275	11.6 (10.8—12.3)	0.8 (0.3—1.4)	12.8 (12.1—13.6)	-2.1 (-2.6—-1.6)	-3.0 (-3.7— -2.2)	<0.0001
Iodine (geometric mean, 95%CI) (μg/d)	273/275	280.7 (255.0—308.9)	104.6% (97.1%—112.7%)	298.0 (270.9—327.7)	95.6% 88.8%—102.9%)	-9.5% (-18.3%—0.2%)	0.055
Iodine (median, IQR) (μg/d)	273/275	275.7 (201.9—360.1)	15.1 (-78.3—105.3)	300.5 (219.2—392.5)	-30.2 (-117.7— 90.2)		
**Completers***							
**Children**							
Salt (mean, 95%CI) (g/d)	135/139	6.8 (6.2—7.4)	1.2 (0.8—1.7)	7.2 (6.6—7.9)	-0.7 (-1.2— -0.2)	-1.9 (-2.6— -1.3)	<0.0001
Iodine (geometric mean, 95%CI) (μg/d)	135/139	162.0 (146.0—180.0)	115.4% (104.9%—126.9%)	173.5 (156.4—192.4)	94.0% (85.6%—103.2%)	-19.3% (-29.4%— -7.8%)	0.002

Iodine (median, IQR) (µg/d)	135/139	160.9 (117.7—208.1)	27.4 (-18.3—76.7)	169.6 (128.5—221.6)	-13.1 (-54.5—37.8)		
**Adults**							
Salt (mean, 95%CI) (g/d)	261/271	11.4 (10.6—12.1)	0.8 (0.2—1.3)	12.7 (11.9—13.5)	-2.2 (-2.7— -1.6)	-3.0 (-3.7— -2.2)	<0.0001
Iodine (geometric mean, 95%CI) (µg/d)	261/270	272.1 (245.3—301.7)	104.7% (96.9%—113.1%)	292.6 (264.1—324.3)	93.1% (86.3%—100.4%)	-11.1% (-20.1%— -1.1%)	0.030
Iodine (median, IQR) (µg/d)	261/270	261.8 (197.8—348.8)	10.7 (-72.8—105.3)	297.7 (213.2—391.8)	-36.5 (-128.4— 88.9)		
**Per protocol population†**							
**Children**							
Salt (mean, 95%CI) (g/d)	132/137	7.0 (6.4—7.6)	1.2 (0.7—1.7)	7.5 (6.9—8.1)	-0.7 (-1.1— -0.2)	-1.9 (-2.6— -1.3)	<0.0001
Iodine (geometric mean, 95%CI) (µg/d)	132/137	169.5 (154.1—186.4)	114.1% (103.7%—125.5%)	179.6 (163.5—197.2)	95.9% (87.3%—105.3%)	-16.8% (-27.2%— -4.9%)	0.007
Iodine (median, IQR) (µg/d)	132/137	166.5 (120.3—216.8)	27.3 (-20.6—79.0)	178.8 (131.3—228.7)	-9.3 (-52.4—39.0)		
**Adults**							
Salt (mean, 95%CI) (g/d)	249/256	11.6 (10.8—12.3)	0.9 (0.3—1.4)	12.9 (12.2—13.7)	-2.1 (-2.6— -1.6)	-3.0 (-3.7— -2.3)	<0.0001
Iodine (geometric mean, 95%CI) (µg/d)	249/255	282.4 (256.5—311.0)	104.3% (96.7%—112.5%)	296.3 (269.3—326.0)	96.1% (89.2%—103.6%)	-8.3% (-17.3%—1.7%)	0.102
Iodine (median, IQR) (µg/d)	249/255	275.8 (203.1—360.1)	15.1 (-78.3—105.3)	300.1 (218.3—391.8)	-30.2 (-117.7— 90.2)		

* Completers refer to the participants who had 24h urine collections both at baseline and end of the trial. † Per protocol population refers to completers with complete 24h urine collections. ‡ Mean and geometric mean were adjusted for stratification variables at randomisation (school location and class size). ‖Adjusted for age, sex, body mass index, stratification variables at randomisation (school location and class size), and indoor and outdoor temperature.

Supplement Table 3. Iodine status assessed by 24h urinary iodine concentration using WHO's criteria, and median 24h urinary iodine concentration and 24h urinary iodine excretion for each category

Urinary iodine (µg/L)	Control						Intervention					
	Baseline			End of trial			Baseline			End of trial		
	N (%)	Iodine (median)		N (%)	Iodine (median)		N (%)	Iodine (median)		N (%)	Iodine (median)	
		(µg/L)	(µg/24h)		(µg/L)	(µg/24h)		(µg/L)	(µg/24h)		(µg/L)	(µg/24h)
**Children**												
**<100** (Iodine deficient)	10 (7.25)	85.07	94.93	10 (7.41)	84.26	114.51	5 (3.55)	86.17	89.84	11 (7.91)	86.65	113.78
**100-199** (Adequate)	55 (39.86)	155.60	139.77	40 (29.63)	152.39	143.31	54 (38.30)	156.60	152.99	48 (34.53)	153.87	125.17
**200-299** (Above requirement)	48 (34.78)	235.92	172.93	52 (38.52)	243.06	184.82	43 (30.50)	248.98	173.10	41 (29.5)	238.24	165.24
**≥300** (Excessive)	25 (18.12)	430.84	249.11	33 (24.44)	371.52	279.42	39 (27.66)	357.28	236.15	39 (28.06)	411.03	260.88
**ALL**	138	204.55	161.73	135	222.49	175.98	141	225.32	166.98	139	217.09	154.82
**Adults**												
**<100** (Iodine deficient)	32 (11.64)	81.31	137.25	38 (14.56)	72.84	149.13	31 (11.19)	79.49	140.35	48 (17.71)	74.71	164.03
**100-199** (Adequate)	121 (44.00)	152.37	240.44	108 (41.38)	147.78	246.58	103 (37.18)	150.95	281.83	116 (42.80)	142.55	250.69
**200-299** (Above requirement)	68 (24.73)	239.54	280.75	72 (27.59)	243.17	353.36	79 (28.52)	246.73	326.61	56 (20.66)	240.86	293.21
**≥300** (Excessive)	54 (19.64)	372.08	420.86	43 (16.48)	351.76	460.61	64 (23.10)	372.21	423.84	51 (18.82)	364.22	535.18
**ALL**	275	188.78	262.11	261	183.55	281.33	277	209.39	297.37	271	176.18	258.54

Supplement Table 4. Comparison of iodine levels observed from 24h urinary iodine with that predicted from salt intake and iodine content in salt

	Control		Intervention	
	**Baseline**	**End of trial**	**Baseline**	**End of trial**
**Children**				
**Observed from 24h urinary measurements**				
**Mean salt (g/d)***	6.8	8.0	7.3	6.6
**Median iodine (μg/d)**	161.7	176.0	167.0	154.8
**Iodine/salt ratio, median (μg/g)**	23.8	24.4	25.0	24.9
**Predicted iodine based on salt intake and iodine content in salt using the minimum level of 18 mg/kg**				
**Predicted iodine (μg/d)**	122.4	144.0	131.4	118.8
**Ratio (predicted/observed)**	0.8	0.8	0.8	0.8
**Adults**				
**Observed from 24h urinary measurements**				
**Mean salt (g/d)***	11.3	12.1	12.6	10.4
**Median iodine (μg/d)**	262.1	281.3	297.4	258.5
**Iodine/salt ratio, median (μg/g)**	24.8	24.8	24.6	26.7
**Predicted iodine based on salt intake and iodine content in salt using the minimum level of 18 mg/kg**				
**Predicted iodine (μg/d)**	203.4	217.8	226.8	187.2
**Ratio (predicted/observed)**	0.8	0.8	0.8	0.7

*adjusted for stratification variables at randomisation (school location and class size).
